# Acute stress blunts prediction error signals in the dorsal striatum during reinforcement learning

**DOI:** 10.1016/j.ynstr.2021.100412

**Published:** 2021-10-27

**Authors:** Joana Carvalheiro, Vasco A. Conceição, Ana Mesquita, Ana Seara-Cardoso

**Affiliations:** a Escola de Psicologia, CIPsi, Universidade do Minho, Campus de Gualtar, 4710-057, Braga, Portugal; b Instituto de Medicina Molecular João Lobo Antunes, Faculdade de Medicina, Universidade de Lisboa, Avenida Professor Egas Moniz, 1649-028, Lisboa, Portugal

**Keywords:** Dopamine, Model-based fMRI, Punishment, Reward, Stress

## Abstract

Acute stress is pervasive in everyday modern life and is thought to affect how people make choices and learn from them. Reinforcement learning, which implicates learning from the unexpected rewarding and punishing outcomes of our choices (i.e., prediction errors), is critical for adjusted behaviour and seems to be affected by acute stress. However, the neural mechanisms by which acute stress disrupts this type of learning are still poorly understood. Here, we investigate whether and how acute stress blunts neural signalling of prediction errors during reinforcement learning using model-based functional magnetic resonance imaging. Male participants completed a well-established reinforcement-learning task involving monetary gains and losses whilst under stress and control conditions. Acute stress impaired participants’ (n = 23) behavioural performance towards obtaining monetary gains (*p* < 0.001), but not towards avoiding losses (*p* = 0.57). Importantly, acute stress blunted signalling of prediction errors during gain and loss trials in the dorsal striatum (*p* = 0.040) — with subsidiary analyses suggesting that acute stress preferentially blunted signalling of positive prediction errors. Our results thus reveal a neurocomputational mechanism by which acute stress may impair reward learning.

## 1 Introduction

Acute stress is ubiquitous in our day-to-day life. Acute stress has major implications for well-being and mental health and, as a consequence, a high societal impact. For example, it is estimated that stress-related disorders, such as depression, anxiety disorders, and alcohol and drug use disorders, affect more than one in six people across European Union countries, and that the total costs of mental health have surpassed €600 billion – or more than 4% of gross domestic product – across the 28 European Union countries (OECD & European Union, 2018). Given that stress has been strongly associated with a broad range of psychopathology (Bogdan and Pizzagalli, 2006; Koob and Volkow, 2016; Mkrtchian et al., 2017; Pizzagalli, 2014; Saal et al., 2003; Sinha, 2007), investigating the mechanisms by which acute stress influences cognition and behaviour is critical not only for understanding the effects of acute stress on day-to-day life, but may also offer important insights into the design of prevention and treatment strategies for individuals with stress-related clinical disorders.

Acute stress is thought to have a deleterious impact on the ability to learn from the outcomes of our choices and to make choices that lead to the most rewarding and least punishing outcomes, which is crucial for adaptive behaviour (Porcelli and Delgado, 2017). A growing body of evidence suggests that reward learning is impaired by acute stress (Berghorst et al., 2013; Bogdan et al., 2011; Bogdan and Pizzagalli, 2006; Carvalheiro et al., 2021; Cremer et al., 2021; de Berker et al., 2016; Ehlers and Todd, 2017; Morris and Rottenberg, 2015; Paret and Bublatzky, 2020), although the evidence for an impairing effect of acute stress on punishment learning is less robust (Carvalheiro et al., 2021; de Berker et al., 2016; Petzold et al., 2010). Yet, surprisingly little is known about the neural mechanisms that underlie the impairing effects of acute stress on reinforcement learning. Here, we use behavioural and model-based functional magnetic resonance imaging (fMRI) (O'Doherty et al., 2007) data to investigate the impact of acute stress on reinforcement learning and the underlying neurocomputational mechanisms.

Reinforcement-learning theory provides a powerful neurocomputational framework to understand how individuals learn to maximise rewards and minimise punishments (Maia and Frank, 2011; Sutton and Barto, 1998). According to reinforcement-learning theory, individuals gradually learn to select more and more often the actions that optimise reinforcements in a given context by learning the values of the executed actions (Maia and Frank, 2011; Sutton and Barto, 1998). Prediction errors — the difference between an experienced and an expected outcome — are used to progressively update the values of the executed actions driving gradual learning (Maia and Frank, 2011; Schultz et al., 1997; Sutton and Barto, 1998). Positive prediction errors indicate that outcomes are better than expected, and negative prediction errors indicate that outcomes are worse than expected (Schultz et al., 1997). Prediction errors can therefore be used to learn which actions are advantageous or disadvantageous. For example, when an action results in an outcome that is better than expected, a positive prediction error occurs, and the value of the action is increased, leading to increased likelihood of selecting that action in the future. Prediction error signals are thought to be encoded in the phasic activity of dopamine neurons (Schultz et al., 1997). Extant evidence indicates that brain areas with dense dopaminergic projections, such as the dorsal striatum and the nucleus accumbens, show activity correlated with prediction errors (O'Doherty et al., 2004; Pessiglione et al., 2006; Valentin & O'Doherty, 2009) and that prediction error signals in the dorsal striatum correlate with behavioural performance efficacy in a reward-based learning task (Schönberg et al., 2007). Indeed, the striatal dopaminergic system seems to be critical for prediction-error-based reward learning (Daw and Tobler, 2014; Glimcher, 2011; Maia and Frank, 2011; Pessiglione et al., 2006).

The striatal dopaminergic system also seems to be particularly sensitive to acute stress (Booij et al., 2016; Cabib and Puglisi-Allegra, 2012; Pruessner et al., 2004). Acute stress elicits a myriad of physiological and functional changes in the brain in response to perceived adverse changes in the environment (de Kloet et al., 2005; Hermans et al., 2014; Joëls and Baram, 2009), including increased dopamine release in the striatum (Abercrombie et al., 1989; Booij et al., 2016; Cabib and Puglisi-Allegra, 2012; Hermans et al., 2014; Joëls and Baram, 2009; Pruessner et al., 2004; Vaessen et al., 2015). Specifically, studies with non-human male animals suggest that acute stress increases aberrant spontaneous phasic-dopamine release (Anstrom et al., 2009; Anstrom and Woodward, 2005; Valenti et al., 2011). Such exaggerated, aberrant spontaneous dopamine release is thought to blunt adaptive phasic dopamine responses that signal positive prediction errors (Daberkow et al., 2013; Maia and Frank, 2017; Werlen et al., 2020) and, more tentatively, negative prediction errors (Maia and Frank, 2017). Thus, stress-induced dopamine aberrant release may lead to impairments in reward learning, and more speculatively in punishment learning.

Extant neural evidence on how acute stress directly affects prediction error signals in the human striatum during reward learning is still scarce (Cremer et al., 2021; Robinson et al., 2013), but indirect neural evidence indicates that women who show the greatest increase in interleukin-6 (an inflammatory marker) in response to a stressor also show the greatest reduction in signalling of prediction errors in the nucleus accumbens during reinforcement learning (Treadway et al., 2017). Moreover, we previously showed, using computational modelling, that acute stress decreases the learning rate for positive prediction errors (i.e., how quickly better-than-expected outcomes are integrated over time) (Carvalheiro et al., 2021), which seems to be consistent with the idea that acute stress might impair reward learning by blunting neural signalling of prediction errors.

In this study, we aimed to investigate the impact of acute stress on striatal prediction error signalling during reinforcement learning. As mentioned above, extant literature suggests that acute stress disrupts reward learning to a larger extent than punishment learning. Thus, given the putative impact of acute stress on aberrant phasic-dopamine release, and the role of adaptive phasic-dopamine responses on prediction error signalling, we predicted that 1) acute stress would impair reward learning and, relatedly, that 2) acute stress would blunt prediction error signals in the striatum during reward learning. Additionally, given that striatal dopamine prediction errors are also implicated in punishment learning (Oleson et al., 2012; Palminteri and Pessiglione, 2017; Seymour et al., 2007), we explored whether and how acute stress would impact punishment learning. Finally, we assessed whether acute stress would preferentially blunt positive or negative prediction error signals during reward and punishment learning.

**Fig. 1:**
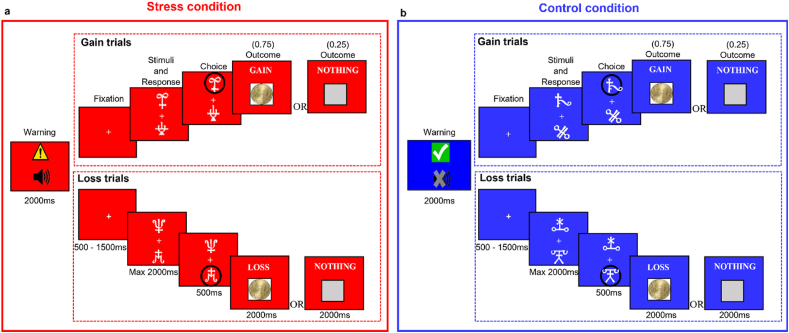
Reinforcement-learning task. Inside the scanner, participants chose between two abstract visual stimuli and observed the outcome of their choice, whilst under acute stress **(a)** and under control conditions **(b)**. In the depicted gain trials, the chosen stimulus was associated with a probability of 0.75 of winning 0.5€ and with a probability of 0.25 of winning nothing. The other (not chosen) stimulus was associated with a reciprocate probability of 0.75 of winning nothing and a 0.25 probability of winning 0.5€. In the depicted loss trials, the chosen stimulus was associated with a probability of 0.75 of losing 0.5€ and with a probability of 0.25 of losing nothing. The other (not chosen) stimulus was associated with a reciprocate probability of 0.75 of losing nothing and a 0.25 probability of losing 0.5€.

Thirty-seven male participants completed an adapted version of a well-established reinforcement-learning task involving monetary gains and losses (Pessiglione et al., 2006) inside the MRI scanner, whilst under acute stress and control conditions (Fig. 1). This reinforcement-learning task disentangles reward from punishment learning and has been used to assess fluctuations in dopamine-prediction errors signals; using this task, combined with pharmacological manipulations of the dopaminergic system, Pessiglione et al. (2006) showed that dopamine-related drugs modulate prediction errors expressed in the striatum during reward (but not during punishment) learning. During the stress condition, participants were exposed to an uncontrollable sound, a constant alarm, which was previously shown to be effective in increasing self-reported stress levels and skin conductance responses rate (Carvalheiro et al., 2021). To check the success of the acute-stress manipulation, we collected self-reported stress levels at the end of each block. Given that we were primarily interested in assessing the effects of acute stress on behaviour and on the neural correlates of prediction errors, we had *a priori* defined that all participants who reported to be non-responsive to the stress manipulation (i.e., who did not report higher stress levels in the stress condition than in the control condition) would be excluded from the main analyses. This resulted in a final pool of 23 participants for behavioural and neuroimaging data analyses. For completeness, we also analysed the data from the total sample, which yielded findings for the impact of acute stress on reward learning consistent with those from the analyses of the aforementioned subsample of interest (see the Supplementary Material for analyses and results concerning the total sample).

To assess whether acute stress impaired reward learning, we inspected the impact of acute stress on task performance. Next, to examine whether and how acute stress blunted signalling of prediction errors in the striatum, we used trial-wise prediction errors, estimated by a well-established reinforcement-learning model (Frank et al., 2007), as parametric modulators of striatal — dorsal striatum and nucleus accumbens — BOLD response at the time of the outcomes in gain (i.e., reward learning) and loss (i.e., punishment learning) trials (Pessiglione et al., 2006).

## 2 Material and methods

### 2.1 Participants

We scanned a total of 42 right-handed male participants with no reported history of neurological or psychiatric disorders. One participant was excluded due to incidental findings and 4 participants were excluded due to technical problems during the scanning session. We assessed whether the stress manipulation increased stress levels by comparing the self-reported stress levels of the remaining 37 participants that completed the task in the stress and control conditions. We established *a priori* that only participants who responded to the stress manipulation would be included in our primary analyses. Self-reported stress levels were higher in the stress condition than in the control condition in 23 participants. Thus, we report results from data analyses of those 23 participants (age range = 18–29 years; *M* = 23.0 years, *SD* = 3.3 years). For completeness, we also analysed the data from the total sample (n = 37); those analyses can be found in the Supplementary Material.

All participants provided their informed consent before the experimental session. All experimental procedures were approved by the Ethics Committee of Hospital of Braga.

### 2.2 Reinforcement-learning task

After a short practice (12 trials) outside the scanner to familiarise participants with the task timings and response keys, participants completed four blocks of an adapted version of a well-established reinforcement-learning task (Pessiglione et al., 2006) whilst inside the scanner (Fig. 1). The task was divided in two runs, each run consisting of a stress block and a control block, totalling four blocks. Stress and control blocks were administered alternately and in a counterbalanced order across the two runs. Each block included three pairs of abstract stimuli, and each pair of stimuli was presented 24 times, totalling 72 trials per block. New abstract stimuli were used in each block. Each pair of stimuli was associated with a valence: one pair of stimuli was associated with gains (gain 0.5€ or no change), a second pair was associated with losses (loss 0.5€ or no change), and a third pair was associated with neutral, or non-financial outcomes (look at a 0.5€ coin or no change). Therefore, each block included stimuli associated with gain, loss and neutral outcomes. The outcome probabilities were reciprocally 0.75 and 0.25 for the stimuli in each of the three pairs. That is, in gain trials, one stimulus was associated with a probability of 0.75 of winning 0.5€ and with a probability of 0.25 of winning nothing (“correct” stimulus), and the other stimulus was associated with a probability of 0.25 of winning 0.5€ and with a probability of 0.75 of winning nothing (“incorrect” stimulus); in loss trials, one stimulus was associated with a probability of 0.75 of losing 0.5€ and with a probability of 0.25 of losing nothing (“incorrect” stimulus), and the other stimulus was associated with a probability of 0.25 of losing 0.5€ and with a probability of 0.75 of losing nothing (“correct” stimulus). We included neutral trials to replicate the task described by Pessiglione et al. (2006) and for fMRI checks of simple contrasts (data not shown), but given that neutral trials were not associated with monetary outcomes (i.e., there were no correct/incorrect responses during neutral trials), participants behavioural performance during these trials was not analysed; regressors for neutral trials were included in the fMRI analyses only for control purposes (i.e., regressors of no interest). On each trial, one pair was randomly presented on the MRI screen, with one stimulus from the pair on the left and the other on the right of a central fixation cross (the stimuli position was counterbalanced across trials). Participants were instructed to choose between the two visual stimuli displayed on the screen to maximise payoffs. Missing choices occurred when participants did not press the response keys within 2000 ms (total of 0.20% missing choices: 8 in the stress condition and 5 in the control condition, in a total of 6624 trials) and were signalled with a “Missed” message (no other outcome was provided). Missing choices were not considered for behavioural data analyses and, where necessary, a regressor for missing choices was included in fMRI analyses for control purposes only (i.e., regressor of no interest). Before starting the task, participants were informed that they would be paid the amount of money obtained during their most profitable block, although they all left with the same fixed compensation (15€) for their participation. The experiment was programmed and presented with Cogent 2000 (http://www.vislab.ucl.ac.uk/cogent.php) implemented in MATLAB R2015a (MathWorks).

### 2.3 Acute-stress manipulation

During the scanning session, participants performed two blocks of the reinforcement-learning task whilst exposed to a stressor (i.e., stress condition) and two blocks without the stressor (i.e., control condition) (Fig. 1). By exposing participants to the stressor during the task, we aimed to make sure that acute stress was contingent on the learning processes. To elicit stress responses, we exposed participants to a predictable, but uncontrollable auditory stimulus: a constant alarm (“Annoying modern office building alarm.wav”, retrieved from freesound.org, and programmed to loop uninterruptedly), played through the scanner with the volume set to the maximum. This uncontrollable sound was always constant and repetitive, to minimise the potential entanglement between stress and distraction, as evidence suggests that unpredictable changes in sound sequences seem to induce distraction more robustly (Hughes, 2014; Parmentier, 2014; Parmentier et al., 2008). Stress blocks were further signalled by a warning sign and a red background (Fig. 1a), and control blocks were signalled by a safe sign and blue background (Fig. 1b).

Stress levels were assessed by asking participants at the end of each block to rate how stressed they felt during that block on a scale of 1 (nothing) to 9 (extremely). We showed in a previous behavioural study that this stress manipulation increased self-reported stress levels and skin conductance responses rate in men (Carvalheiro et al., 2021).

### 2.4 Task-performance analyses

To examine the impact of acute stress on behavioural choice performance during the reinforcement-learning task, we applied a generalized linear mixed-effects (glme) model to participants' trial-by-trial choice data (with correct and incorrect choices coded as 1 and 0, respectively). We used a “logit” link function to account for the binomial distribution of the data. We included as predictor variables in the glme model: condition (stress or control), valence (gain or loss), block number (1 or 2), trial number (1–24), and the interaction of interest (condition × valence). The glme included a fixed intercept, as well as random intercepts for each participant. We fitted the glme model to the behavioural data using MATLAB's *fitglme* function and performed planned post-hoc analyses via contrast matrices using MATLAB's *coefTest* function.

### 2.5 fMRI data acquisition and preprocessing

A Siemens Verio 3T MRI scanner at the Clinical Academic Center – Braga with a 32-channel head coil was used to acquire a 5.5 min 3D T1-weighted anatomical scan and multislice T2*-weighted echo planar images (EPIs) with BOLD contrast. The T2* EPI sequence used the following acquisition parameters: field of view = 200 × 200 mm, matrix size = 66 × 66 mm, interleaved slice order acquisition, 42 slices with slice thickness of 3 mm with no gap between slices, flip angle of 60°, echo time of 22 ms, and repetition time of 2000 ms. Functional task-related data were acquired in two runs, separated by a short break during which participants remained inside the scanner in the same position. Fieldmaps were acquired for use in the unwarping stage of data preprocessing. Imaging data were analysed using SPM12 (www.fil.ion.ucl.ac.uk/spm). Data preprocessing followed a standard sequence: the first five volumes were discarded, and data were realigned to the sixth volume, unwarped using a fieldmap (normalised to the Montreal Neurological Institute, MNI, template), and coregistered to the participant's own anatomical image. The anatomical images were normalised using a unified segmentation procedure (Ashburner and Friston, 2005), combining segmentation, bias correction, and calculation of the wrapping or distortions needed to map the anatomical image into Montreal Neurological Institute space (i.e., deformation fields), and then applying these warps to the EPI data. The voxel size was resampled to 1.5 × 1.5 × 1.5 mm. Last, a Gaussian kernel of 8 mm FWHM was applied to smooth the images spatially.

### 2.6 fMRI data analyses

#### 2.6.1 Primary general linear model

The primary fMRI analyses were based on a single general linear model, as in previous studies that used a similar reinforcement-learning task (Kumar et al., 2018; Pessiglione et al., 2006; Treadway et al., 2017). Each trial was modelled as having two time points: stimuli and outcome onsets. Note that, although our analyses focused on the prediction errors at the onset of outcomes, the onsets of stimuli were also modelled, to account for likely shared variance between BOLD signals at the time of the stimuli and outcomes. Separate regressors were created for the 6 types of stimuli [2 conditions (stress/control) × 3 valences (gain/loss/neutral)] and the 6 types of outcomes [2 conditions (stress/control) × 3 valences (gain/loss/neutral)] in each run (see Supplementary Fig. 5 for an example of a first-level design matrix); the regressors were modelled as stick functions and convolved with SPM's canonical hemodynamic response function (Pessiglione et al., 2006). Each time point was regressed with a parametric modulator, separately for gain and loss trials: stimuli onset was modulated by the value of the chosen option, $Q_{chosen}\left(t\right)$; and, importantly, outcome onset was modulated by the prediction error, δ(t). Such values and predictions errors were estimated trial-wise using a well-established reinforcement-learning model (Frank et al., 2007). Briefly, in this reinforcement-learning model, the value of the chosen stimulus, *Q*_*chosen*_, is updated on each trial, *t*, according to the following learning rule: $Q_{chosen}\left(t+1\right)=\;Q_{chosen}\left(t\right)+\;\alpha *\delta \left(t\right)$. The prediction error, δ(*t*), is the difference between the actual and the expected outcome: $\delta \left(t\right)=r\left(t\right)-Q_{chosen}\left(t\right)$, where the reinforcement *r*(*t*) is either 0.5, 0, or −0.5. The used reinforcement-learning model included separate learning rates for positive (*α*^*+*^) and negative (*α*^*-*^) prediction errors to account for the differential neural signalling of positive and negative prediction errors (Maia and Frank, 2011; Schultz et al., 1997). The reinforcement-learning model also included the inverse temperature parameter, *β*, which controls the amount of noise in choice selection (see Supplementary Material for a detailed description of the reinforcement-learning model). Values and prediction errors were estimated using the parameters *α*^±^ and *β* estimated for each subject in each condition and used as separate parametric modulators of neural activity at the time of stimuli and outcomes, respectively, either in gain or loss trials, in each condition. We also included an additional regressor to model missed trials, when participants did not select one of the two symbols and there was no outcome. For participants with visible headmotion in a particular scan (scans with >1 mm or 1° movement relative to the next) an extra regressor was included. Those images were removed and replaced with an image created by interpolating the two adjacent images to prevent distortion of the between-subjects mask (seven participants with visible headmotion; less than 1% of the total time series for each of them). Six headmotion parameters modelled the residual effects of headmotion. Data were high-pass filtered at 128s to remove low-frequency drifts, and the general linear model included an AR(1) autoregressive function to account for autocorrelations intrinsic to the fMRI time series.

Our primary analyses focused on prediction errors at outcome. First-level contrast images were calculated by applying appropriate linear contrasts to the parametric modulators of interest — prediction errors — and were entered into second-level analyses. Second-level one-sample *t*-tests were conducted for each contrast using the summary-statistics approach to random-effects analysis. Regions of interest (ROI) analyses in the dorsal striatum and nucleus accumbens were conducted using an initial threshold of *p* < 0.001 (uncorrected) and responses were considered significant if they survived voxel-level small-volume family-wise error correction (SVC-FWE) at *p* < 0.05. The *a priori* ROIs — dorsal striatum and nucleus accumbens — were anatomically defined using masks. Specifically, a bilateral mask for the dorsal striatum was defined using a conjunction of the left and right putamen and caudate from the automated anatomical labelling (AAL) atlas. A bilateral mask for the nucleus accumbens was defined using a conjunction of the left and right nucleus accumbens from the Individual Brain Atlases using Statistical Parametric Mapping (IBASPM). As the bilateral nucleus accumbens mask had a slight overlap with the dorsal striatum mask, we subtracted the mask of the nucleus accumbens from the dorsal striatum mask. The atlases and the conjunctions were implemented using the WFU PickAtlas Toolbox in SPM12. Individual BOLD estimates (i.e., regression slopes) of prediction error parametric modulators were extracted from significantly activated clusters using the MarsBaR toolbox (Brett et al., 2002). For completeness, we also explored the impact of acute stress at the whole-brain level. Regions are reported at FWE corrected *p* < 0.05 at cluster level following an initial uncorrected threshold of *p* < 0.001 (minimum of 10 contiguous voxels).

#### 2.6.2 Subsidiary general linear models

To better understand the impact of acute stress on prediction error signals, we generated two subsidiary general linear models. Note that these two subsidiary models did not include any parametric modulators, as our purpose was to visualise how the BOLD response varied along different magnitudes of prediction errors.

For the first subsidiary model, we split prediction errors into four equally sized bins. The boundaries of the bins did not differ significantly between the stress and control conditions, in gain (all *p* > 0.071, paired *t*-tests) nor in loss (all *p* > 0.13, paired *t*-tests) trials (Supplementary Table 2). Specifically, this first subsidiary general linear model included separate regressors for trials corresponding to each bin, in each valence (gain and loss) and condition (stress and control), modelled at the stimuli and outcome onset (as in the primary general linear model) resulting in thirty-two regressors, plus regressors for neutral trials in each condition, missing trials (if applicable) and headmotion (and visible headmotion, if applicable), for each run.

For the second subsidiary model, we split prediction errors into negative and positive. This model included separate regressors for trials corresponding to negative and positive prediction errors, in each valence (gain and loss) and condition (stress and control), modelled at the stimuli and outcome onset (as in the primary general linear model) resulting in sixteen regressors, plus regressors for neutral trials in each condition, missing trials (if applicable) and headmotion (and visible headmotion, if applicable), for each run.

In both subsidiary models, the average BOLD estimates at the outcome onset (when prediction errors occur) were extracted from the significant dorsal striatum cluster identified in the primary general linear model, using the MarsBaR toolbox (Brett et al., 2002).

## 3 Results

### 3.1 Behavioural analyses

#### 3.1.1 Manipulation check

**Fig. 2:**
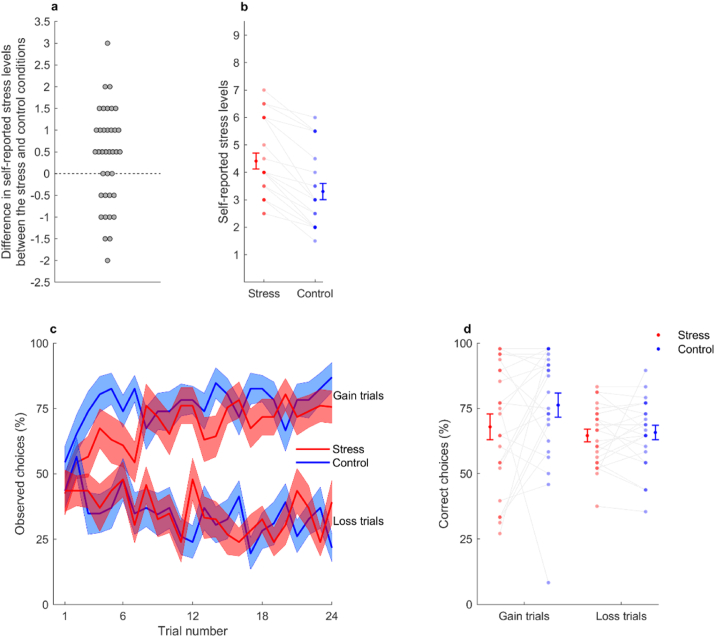
Manipulation check and task performance. **(a)** Difference in self-reported stress levels between the stress and control conditions (averaged across blocks). Each grey dot represents a participant (n = 37). Participants who reported higher stress levels in the stress than in the control condition correspond to the dots above the horizontal dashed line (n = 23)**. (b)** Self-reported stress levels in stress (red) and control (blue) conditions (averaged across blocks) in the pool of participants that reported higher stress levels in the stress than in the control condition (n = 23). **(c)** Learning curves represent the trial-by-trial percentage of participants (n = 23) who chose the correct gain stimulus (associated with a probability of 0.75 of winning 0.5€; upper part of the graph) and the incorrect loss stimulus (associated with a probability of 0.75 of losing 0.5€; lower part of the graph), in the stress and control conditions. Each central line represents the mean and each filled area the ±standard error of the mean. **(d)** Percentage of correct choices per participant (n = 23) in gain and loss trials, across the stress and control conditions (averaged across blocks). In graphs b and d, connected dots represent data points from the same participant, and more transparent (opaque) dots represent less (more) overlapping data points; error bars displayed on the sides of those scatter plots indicate the mean ± standard error of the mean. (For interpretation of the references to colour in this figure legend, the reader is referred to the Web version of this article.)

First, we computed the difference in self-reported stress levels between the stress and control conditions in the total sample (n = 37). Twenty-three participants reported higher stress levels in the stress condition than in the control condition (Fig. 2a). Then, we conducted analyses of variance (ANOVAs) with condition (stress and control) and block (1 and 2) as within-subject factors in those 23 participants (see Supplementary Material for manipulation-check analyses of the total sample). Self-reported stress levels differed significantly between conditions (*F*_1,22_ = 69.28, *p* < 0.001, *ƞ*^*2*^ = 0.76) (Fig. 2b), but there was no main effect of block (*F*_1,22_ = 0.008, *p* = 0.93, *ƞ*^*2*^ = 0) and the condition × block interaction was also non-significant (*F*_1,22_ = 1.21, *p* = 0.28, *ƞ*^*2*^ = 0.052). This suggests that self-reported stress levels increased with the acute-stress manipulation and remained stable across blocks within each condition for these participants. Participants whose self-reported levels of stress did not increase with the acute stress induction were excluded from the following analyses (but see Supplementary Material for total sample analyses).

#### 3.1.2 Task performance

To assess whether acute stress blunted reward learning, we examined the impact of acute stress on choice performance during the reinforcement-learning task (Fig. 2c). We used a generalized linear mixed-effects (glme) model, which accounted for the binomial distribution of the trial-by-trial data (correct or incorrect responses). The glme model included condition (stress or control), valence (gain or loss), block number (1 or 2), trial number (1–24), and the interaction of interest (condition × valence) as predictor variables. We found a significant condition × valence interaction (*β* = −0.39, *p* = 0.0038, 95% CI = [−0.66, −0.13]) (Fig. 2d). Planned post-hoc analyses showed that under stress, comparatively to the control condition, participants performed significantly worse when learning to obtain gains (*F*_1, 4400_ = 20.23, *p* < 0.001), but not when learning to avoid losses (*F*_1, 4400_ = 0.32, *p* = 0.57). Additionally, we identified one participant who showed an abnormally low percentage of correct answers during trials in the control condition (8.3%, Fig. 2d), which might reflect aberrant learning (although the overall performance of this participant during the whole task was above chance levels). Therefore, we repeated the analyses excluding this participant and confirmed that the significance of the interaction remained unchanged (condition × valence interaction: *β* = −0.69, *p* < 0.001, 95% CI = [−0.97, −0.41]).

In sum, acute stress selectively impaired choice performance towards monetary gains during the reinforcement-learning task.

### 3.2 fMRI analyses

#### 3.2.1 Primary general linear model

##### 3.2.1.1 Prediction error signals in the striatum

To examine the impact of acute stress on prediction error signalling in the striatum during reinforcement learning, we generated a primary fMRI general linear model that included prediction errors as parametric modulators of BOLD response in the striatum (dorsal striatum and nucleus accumbens) at the time of the outcomes in gain and loss trials, in the stress and control conditions (see “Material and methods” and Supplementary Fig. 5 for further details on the primary general linear model). Prediction errors were estimated in the stress and control conditions using a reinforcement-learning model that has been extensively used to investigate the behavioural and neural impact of pharmacological manipulations and genetic variations in the dopaminergic system in humans (Diederen et al., 2017; Doll et al., 2011; Frank et al., 2007; Frank and Fossella, 2011; Grogan et al., 2017; Rutledge et al., 2009). The computational modelling methods, results and respective discussion can be found in the Supplementary Material (see “Computational modelling” section). Parametric analyses incorporating prediction errors allow a more precise estimation of how brain activity fluctuates during learning compared to examination of outcome-associated activation alone (O'Doherty et al., 2007).

As expected, we observed that BOLD response in the dorsal striatum and nucleus accumbens — regions consistently shown to respond to unexpected rewards and punishments (Fouragnan et al., 2018; Garrison et al., 2013; Pessiglione et al., 2006) — scaled parametrically with the magnitude of prediction errors at the time of the outcomes, during gain and loss trials, in both conditions. Specifically, we identified a positive parametric modulation of prediction errors in the dorsal striatum bilaterally (i.e., the magnitude of the prediction errors correlated positively with BOLD response in this region on a trial-by-trial basis), during gain and loss trials, in both conditions [all *Z* > 4.12, *p* < 0.05, voxel-level small-volume family-wise error corrected (SVC-FWE)]. We also found a positive parametric modulation of prediction errors in the nucleus accumbens during gain trials, in both conditions, and during loss trials in the control condition (all Z > 3.63, *p* < 0.05, SVC-FWE; see Supplementary Table 1 for whole-brain and all SVC-FWE results).

##### 3.2.1.2 Effects of acute stress on prediction error signals in the striatum

After confirming that striatal activity scaled parametrically with the magnitude of prediction errors, we inspected whether acute stress affected prediction error signals in the striatum. To examine whether acute stress would blunt signalling of prediction errors in the striatum during reward learning, first we tested the main effect of stress using the control > stress contrast for the parametric modulation of prediction errors at the time of the outcomes delivered across gain and loss trials in each condition. A significant main effect would mean that acute stress decreased prediction error signals across gain and loss trials. Second, we tested the contrast for the condition (stress or control) × valence (gain or loss) interaction. A significant interaction would mean that acute stress affected prediction error signals differently in gain and loss trials.

**Fig. 3:**
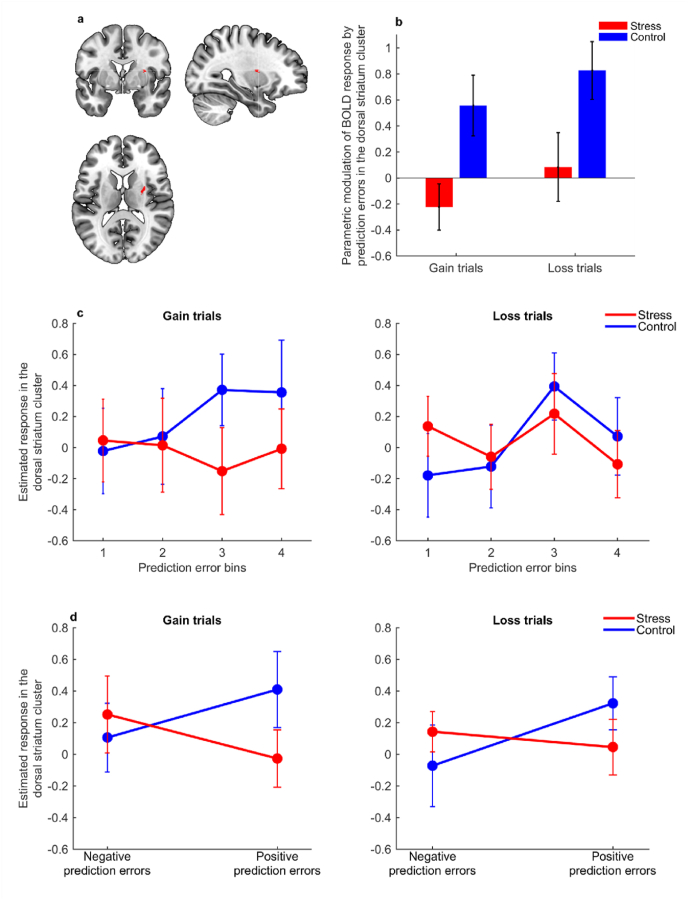
Effects of acute stress on prediction error signalling in the dorsal striatum. **(a)** Cluster in the dorsal striatum where the modulation of prediction errors at the time of the outcome was significantly decreased in the stress condition compared with the control condition (SVC-FWE, *p* < 0.05). **(b)** Bars depict parameter estimates (i.e., regression slopes) for the BOLD response at the dorsal striatum cluster [from (a)] modulated by trial-by-trial prediction errors, in gain and loss trials, across the stress (red) and control (blue) conditions (n = 23). **(c, d)** Graphs represent the modulation of BOLD response by prediction errors at the time of the outcome in gain (left) and loss (right) trials, in the dorsal striatum cluster identified in the primary general linear model [depicted in (a)], in the stress (red) and control (blue) conditions (n = 23). BOLD response estimates within the dorsal striatum cluster were extracted for each participant. Error bars indicate the mean ± standard error of the mean. In **(c)**, data for illustrative graphs were derived from a subsidiary model where trials were divided into quartiles of magnitude of prediction errors (with the lowest and highest magnitudes corresponding to bins 1 and 4, respectively). In **(d)**, the plotted data were obtained from a second subsidiary model where trials were divided into negative and positive prediction errors. (For interpretation of the references to colour in this figure legend, the reader is referred to the Web version of this article.)

The contrast control > stress showed a significant main effect of stress on the parametric modulation of prediction errors in the dorsal striatum ([*x* = 32, *y* = 0, *z* = 12], *Z* = 4.08, *k* = 26, *p* = 0.040, SVC-FWE) (Fig. 3a), meaning that prediction error signals were decreased in the stress condition compared with the control condition, both in gain and loss trials (Fig. 3b). Confirmatory one sample *t*-tests comparing the parameter estimates (i.e., the regression slopes from the primary general linear model) extracted from the identified dorsal striatum cluster against zero, indicated that the parametric modulation of prediction errors was significantly higher than zero in the control condition, both for gain and loss trials (both *p* < 0.023), but not in the stress condition (both *p* > 0.33) (Fig. 3b). We did not observe any significant responses for the parametric modulation of prediction errors in the nucleus accumbens for the control > stress contrast (nor for the inverse contrast stress > control).

For the contrast that tested the condition × valence interaction, we did not find any significant activations in the dorsal striatum nor in the nucleus accumbens. This non-significant interaction, together with the significant main effect described above, indicates that acute stress blunted prediction errors in the dorsal striatum both in gain and loss trials.

For completeness, we also explored whether acute stress affected prediction error signals in other brain areas by searching for significant activations in the whole brain. For the contrast control > stress, no significant activations were found (FWE corrected *p* < 0.05 at cluster level following an initial uncorrected threshold of *p* < 0.001). For the contrast that tested the condition × valence interaction we found significant activations in two temporal clusters (Supplementary Fig. 6a), one of which extended to the insula ([−47 6–11], *Z* = 4.23, *k* = 218, *p* = 0.015, FWE corrected *p* < 0.05 at cluster level following an initial uncorrected threshold of *p* < 0.001), which has been associated with prediction error signals during punishment learning (Garrison et al., 2013; Pessiglione et al., 2006). Within this cluster, we found a positive parametric modulation of prediction errors during gain trials in the control condition, but in the stress condition such modulation was negative and significantly decreased compared with the control condition (*t*_22_ = 3.2, *p* = 0.0041); during loss trials, we found a negative parametric modulation of prediction errors in the control condition, but in the stress condition such modulation was positive and significantly increased compared with the control condition (*t*_22_ = 2.98, *p* = 0.0069) (Supplementary Fig. 6b).

###### 3.2.1.2.1 Associations between prediction error signals in the striatum under acute stress and self-reported stress levels

To assess whether the identified modulation of prediction errors under stress varied with self-reported stress responsivity, we correlated the parameter estimates extracted from the dorsal striatum cluster with the difference in self-reported levels between the stress and control conditions. We found a trend towards a negative association between the parametric modulation of prediction errors during gain trials in the stress condition and self-reported stress responsivity (r_*s*_ = −0.40, *p* = 0.059); moreover, such negative association was stronger and significant in the total sample (r_*s*_ = −0.52, *p* < 0.001; Supplementary Fig. 4), which allowed us to obtain a more complete picture of how prediction error signalling varied across a larger range of self-reported stress responsivity levels. These results indicate that participants who reported the greatest increase in stress levels in response to the acute stressor showed the greatest reduction in signalling of prediction errors in the dorsal striatum during gain trials under acute stress.

Overall, we found that the BOLD response in the dorsal striatum scaled parametrically with the magnitude of prediction errors during reward and punishment learning, both under acute stress and control conditions. More importantly, we found that signalling of prediction errors in the dorsal striatum was blunted by acute stress. Additionally, such blunting was related to self-reported stress responsivity levels.

#### 3.2.2 Subsidiary general linear models

To illustrate the effect of acute stress on the parametric modulation of prediction errors in the dorsal striatum described in the previous section, we conducted a subsidiary general linear model. In this subsidiary model, we extracted estimates of BOLD response across trials of different categories of prediction error magnitudes from the dorsal striatum cluster that we had previously identified (cluster represented in Fig. 3a). Specifically, in this subsidiary model, trials from each condition (i.e., stress and control) and valence (i.e., gain and loss) were further divided into four bins corresponding to quartiles of magnitude of prediction errors (see Supplementary Table 2 for median and boundaries of each bin). Parameter estimates of BOLD response at the outcome onset were extracted from the dorsal striatum cluster for each subject (see “Material and methods” for full description) and plotted to illustrate the variation in the BOLD response in the dorsal striatum cluster along the magnitude of prediction errors. The blunting effect of stress on prediction error signals in the dorsal striatum (both in gain and loss trials) seemed to be mostly driven by decreased signalling of prediction errors of higher magnitude (Fig. 3c). Relatedly, the 1st bin and 4th bins roughly corresponded to negative and positive prediction errors, respectively (Supplementary Table 2), suggesting that acute stress mostly decreased positive prediction error signals.

To further explore whether acute stress had preferentially blunted signalling of positive prediction errors during reward and punishment learning, we conducted an additional parametric modulation model similar to the primary general linear model, but this time we modelled positive and negative prediction errors separately for either gain or loss trials. We did not find any parametric modulations of the dorsal striatum or nucleus accumbens response by positive nor negative prediction errors, likely due to reduced variance within each parametric modulator. Therefore, and for completeness, we performed a second subsidiary general linear model (see “Material and methods” for full description). In this subsidiary model, trials from each condition (i.e., stress or control) and valence (i.e., gain or loss) were further divided according to the prediction error valence (i.e., positive or negative). We extracted estimates of BOLD response in the dorsal striatum cluster (cluster represented in Fig. 3a) at the outcome onset, when positive or negative prediction errors occurred, and performed an ANOVA on those estimates derived from the subsidiary model. We found a significant condition × prediction error valence interaction (*F*_1,22_ = 8.84, *p* = 0.007, *η*^2^ = 0.29) (Fig. 3d), indicating that acute stress differently affected positive and negative prediction errors, both in gain and loss trials (the condition × prediction error valence × trial valence interaction was non-significant, *F*_1,22_ = 0.047, *p* = 0.83, *η*^2^ = 0.0020). Post-hoc planned comparisons were non-significant, but inspection of effect sizes suggested that acute stress decreased positive prediction error signals (paired t-tests in gain trials: *t*_22_ = −1.36, *p* = 0.19, Cohen's *d* = −0.28; in loss trials: *t*_22_ = −1.12, *p* = 0.27, Cohen's *d* = −0.23) to a larger extent than negative prediction errors (paired t-tests in gain trials: *t*_22_ = 0.48, *p* = 0.64, Cohen's *d* = 0.10; in loss trials: *t*_22_ = 0.86, *p* = 0.40, Cohen's *d* = 0.18) (Fig. 3d), in line with the previous subsidiary analysis of prediction error bins depicted in Fig. 3c. Additionally, we repeated all the fMRI analyses excluding the participant who responded correctly in only 8.3% of gain trials during the control condition, and the interpretation of the results remained unchanged (see Supplementary Fig. 7).

Taken together, subsidiary data suggests that acute stress mostly decreased positive prediction errors signals.

## 4 Discussion

### 4.1 Effects of acute stress on prediction error signals during reinforcement learning

Acute stress is ubiquitous in modern day-to-day life and previous studies have found that it impacts reinforcement learning. Yet, the mechanisms that underlie the impact of acute stress on reinforcement learning are still poorly understood. Acute stress alters human striatal dopaminergic functioning (Booij et al., 2016; Cabib and Puglisi-Allegra, 2012; Pruessner et al., 2004; Vaessen et al., 2015), and dopaminergic functioning plays a key role in signalling of prediction errors (Daw and Tobler, 2014; Glimcher, 2011; Maia and Frank, 2011; Pessiglione et al., 2006) — the result of a positive or negative difference between obtained and expected outcomes — which drive reward and punishment learning. Thus, we set out to test whether acute stress impaired reward learning by blunting prediction error signals in the striatum and further explored the putative impact of acute stress on punishment learning.

In line with extant literature (Berghorst et al., 2013; Bogdan et al., 2011; Bogdan and Pizzagalli, 2006; Cremer et al., 2021; de Berker et al., 2016; Ehlers and Todd, 2017; Morris and Rottenberg, 2015; Paret and Bublatzky, 2020), we replicated our previous finding that acute stress impairs reward-seeking performance (Carvalheiro et al., 2021). We had originally shown this in a larger, independent university male sample (n = 62), but we now show additionally that this behavioural impairment was accompanied by blunted signalling of prediction errors in the dorsal striatum. Although neural data indicated that acute stress also blunted prediction error signals in the dorsal striatum during punishment learning, this was not observed at the behavioural level. Relatedly, subsidiary analyses suggested that acute stress blunted positive prediction error signals preferentially, which might explain the differential impact of acute stress on reward and punishment learning.

Our finding that acute stress blunted signalling of prediction errors — and mostly positive prediction errors — in the dorsal striatum during reward learning is consistent with a neurobiological framework of stress-induced dopamine disruptions. Prediction errors are encoded in phasic activity of dopaminergic neurons (Daw and Tobler, 2014; Glimcher, 2011; Schultz et al., 1997). Phasic bursts of dopaminergic neurons are thought to adaptively encode positive prediction errors, whereas dopamine dips have been associated with the adaptive encoding of negative prediction errors (Daw and Tobler, 2014; Schultz et al., 1997). However, phasic-dopamine responses do not seem to be always adaptive, and there is evidence that dopamine can be phasically released in an aberrant spontaneous manner (Grace, 2016; Maia and Frank, 2017; Sulzer et al., 2016; Wightman et al., 2007). Relatedly, studies with non-human male animals suggest that acute stress induces aberrant spontaneous dopamine release (Anstrom et al., 2009; Anstrom and Woodward, 2005; Valenti et al., 2011). It is therefore possible that, if acute stress increases aberrant spontaneous phasic-dopamine release, then phasic dopamine release that signals positive prediction errors is less easily differentiated from background fluctuations in dopamine levels, resulting in a low signal to noise ratio (Grace, 2016; Sulzer et al., 2016).

In addition, if there is increased aberrant spontaneous release of dopamine, then less dopamine may be available to be released from dopaminergic neurons when positive prediction errors occur (Sulzer et al., 2016). Thus, stress-induced aberrant dopamine release may indirectly or directly blunt positive prediction errors that signal unexpected rewards (Daberkow et al., 2013; Maia and Frank, 2017; Werlen et al., 2020), resulting in impaired reward learning. Furthermore, we previously showed, using computational modelling, that acute stress decreases the learning rate for positive prediction errors (Carvalheiro et al., 2021), which is in striking agreeement with our neuroimaging data. Stress-induced blunted prediction errror signals — and preferentially positive prediction errors — in the dorsal striatum during reward learning might explain why individuals have difficulties in updating their behaviour in response to unexpected rewards when under acute stress.

Previous work suggests that punishment learning may not be affected by acute stress to the same extent as reward learning is (Berghorst et al., 2013; Carvalheiro et al., 2021; Porcelli and Delgado, 2017). In this study we did not find evidence of a behavioural impairment of acute stress on punishment learning. Although our neuroimaging data initially suggested an effect of acute stress on the signalling of prediction errors in the dorsal striatum during punishment learning, further subsidiary analyses indicated that the main effect of stress on prediction errors seems to be mostly explained by decreased signalling of positive prediction errors. It is thus possible that acute stress compromises the ability to use dopamine phasic bursts that signal positive prediction errors but not to use dopamine dips that encode negative prediction errors. Indeed, empirical evidence suggests that aberrant spontaneous dopamine release decreases striatal adaptive phasic dopamine responses that signal positive prediction errors (Daberkow et al., 2013; Werlen et al., 2020), and, only more speculatively, negative prediction errors (Maia and Frank, 2017). Although positive prediction errors can also occur during punishment learning, in simple reinforcement-learning tasks, such as ours, punishment learning seems to be largely driven by negative prediction errors (Palminteri and Pessiglione, 2017). Consequently, stress-induced disruptions in positive prediction errors might not necessarily be reflected in impaired learning from punishments. Finally, non-dopaminergic mechanisms may also be involved in punishment learning (Boureau and Dayan, 2011; Daw et al., 2002), which might partially explain why previous studies using similar reinforcement-learning tasks also did not find significant effects of pharmacological manipulations of the dopaminergic system on punishment learning (Eisenegger et al., 2014; Pessiglione et al., 2006).

The dorsal striatum has been associated with reward-based action selection (O'Doherty et al., 2004; Schönberg et al., 2007) — and is thus thought to play a key role in instrumental learning tasks, such as ours, by maintaining information about action-contingent response-reward associations to guide future choices based on the outcomes of past ones — whereas the ventral portion of the striatum, the nucleus accumbens, has been more implicated in classical conditioning (O'Doherty et al., 2004). By blunting prediction error signals in the dorsal striatum, acute stress may thus impair learning of stimulus-response-reward associations, which are crucial to perform our reinforcement-learning task. We did not find evidence for an effect of acute stress on prediction errors within the nucleus accumbens. Although previous studies suggest that acute stress affects prediction error signals in the nucleus accumbens (Robinson et al., 2013; Treadway et al., 2017), other recent study has found that acute stress affects prediction error signals in the dorsal striatum (putamen) but not in the nucleus accumbens (Cremer et al., 2021). In addition, although there is considerable evidence from non-human animal studies that acute stress affects dopaminergic functioning in the ventral tegmental area (Anstrom et al., 2009; Anstrom and Woodward, 2005; Valenti et al., 2011), which densely projects to the nucleus accumbens, it is plausible that different dopaminergic subpopulations are affected to a different extent during stress depending on the task being executed, as well as on the duration and type of the stressor (Holly and Miczek, 2016). Our results provide evidence that acute stress blunts prediction error signals in the dorsal striatum, but different stressors may affect distinct regions of the striatum and the functions they support differently.

Additionally, our exploratory whole-brain analyses suggest that acute stress might affect other brain areas, such as temporal areas and the insula. The anterior insula has been associated with prediction error signals in aversive contexts (Fouragnan et al., 2018; Garrison et al., 2013; Pessiglione et al., 2006), but it is also thought to play an important role in signalling salience (Rutledge et al., 2010) or surprise of an outcome (i.e., unsigned prediction errors) (Fouragnan et al., 2018). Our whole-brain analyses, focused on a temporal cluster that extended to the insula in the control condition, pointed to a positive modulation of prediction errors (i.e., the BOLD response increased as the magnitude of prediction errors increased) during reward learning, but to a negative modulation of prediction errors (i.e., the BOLD response increased as the magnitude of prediction errors decreased) during punishment learning, which seems consistent with a role of the insula in coding salience (Rutledge et al., 2010); moreover, acute stress seems to affect these differential modulations during reward and punishment learning. Thus, it is possible that acute stress interferes not only with the parametric modulation of prediction errors in the dorsal striatum, but also with computations of salience in other brain areas. Further studies are required to better understand the impact of acute stress on the neurocomputational mechanisms of classical and instrumental reward and punishment learning and could focus not only on the striatum, but also on other brain areas, such as the anterior insula.

Our findings suggest that, when under acute stress, the value of actions that resulted in past rewarding outcomes is disrupted — due to blunted signalling and integration of positive prediction errors — and it is less likely that such action is chosen in the future. It is not well-known yet how this might translate into real-life settings, but it can be speculated that under stress people might not engage so much in novel pleasant actions, such as starting a new hobby, because the positive outcomes that result from those actions are more poorly signalled, decreasing the value of the actions and the likelihood of selecting them again in the future. This can be partially linked with experimental evidence that acute stress increases habitual behaviours (Porcelli and Delgado, 2017; Schwabe and Wolf, 2009; Smeets et al., 2019), which are characterised by a loss of sensitivity to the rewarding outcomes of actions and do not require update of reward expectations (Lingawi et al., 2016). In other words, it is possible that acute stress interferes with selection of actions that implicate update of expectations via prediction errors, and particularly positive prediction errors. Interestingly, it has been argued that effective psychotherapies, such as cognitive behavioural therapy, work by challenging and updating such expectations via prediction errors to induce new learning and behavioural change (Nair et al., 2020; Queirazza et al., 2019; Papalini et al., 2020). Therefore, our findings might have implications for the design of psychological interventions focused on targeting the negative impact of acute stress on reward learning.

### 4.2 Limitations

In this study, we induced acute stress in participants, using a repetitive and uncontrollable sound. This manipulation was previously validated outside the scanner [for a thorough discussion about the choice and validation of the stressor see Carvalheiro et al. (2021)]. In the current study, the stressor increased self-reported stress levels, although to a lesser extent than in our previous experiment. One potential explanation for this discrepancy is that, inside the scanner, the stressor was not as salient as it was outside the scanner. We used additional visual cues as warning signals and coloured backgrounds to amplify the effects of the stress manipulation. Importantly, our data showed that, under acute stress, signalling of prediction errors during reward learning decreased as the difference in self-reported stress between the stress and control conditions (i.e., stress responsivity) increased, suggesting that prediction error signals are not only affected by the presence of acute stress, but also that these neural signals may vary negatively with perceived changes in stress levels.

In the present work, we were unable to analyse physiological responses due to technical limitations. However, we had previously shown that our stress manipulation increased skin conductance response rate (Carvalheiro et al., 2021). The inclusion of physiological measures, such as skin conductance, heart rate and cortisol, in future research would be useful to further validate the efficacy of our stress manipulation, while providing valuable information on individual differences in stress responsivity.

The reinforcement-learning model used in this work, which includes separate learning rates for positive and negative prediction errors (Frank et al., 2007), is well-established and has been extensively used to investigate the cognitive and behavioural impact of pharmacological manipulations and genetic variations in the dopaminergic system in humans (e.g., Diederen et al., 2017; Doll et al., 2011; Frank and Fossella, 2011; Frank et al., 2007; Grogan et al., 2017; Rutledge et al., 2009). However, given the inter-individual variability in the extent to which participants learned how to seek rewards and avoid punishments under acute stress and control conditions, it is possible that behavioural data from some participants might be better fit with alternative reinforcement-learning models (Wilson and Collins, 2019).

Our fMRI study was not designed to test specific neural effects of acute stress on positive and/or negative prediction errors and can only provide tentative evidence for this association. We built subsidiary fMRI models to assess potential effects of acute stress that varied according to the sign of prediction error, and we conducted exploratory analyses on the estimates obtained from those models without correction for multiple testing. Although our findings are preliminary, they seem relevant and could be used to generate and test specific hypothesis in future studies.

fMRI studies have consistently shown that the reinforcement-learning task used in our work captures BOLD response in the striatum associated with prediction error signals (Pessiglione et al., 2006; Kumar et al., 2018; Lefebvre et al., 2017; Palminteri et al., 2015; Treadway et al., 2017; Voon et al., 2010). However, to better argue for a robust effect of acute stress on striatal prediction errors and to make inferences about individual differences, further investigations could benefit from examining the reliability of our task-fMRI measures (Elliott et al., 2020).

To avoid potential confounding effects of menstrual-cycle-dependent variation on stress responsivity (Ossewaarde et al., 2010) as well as on reward and punishment learning (Diekhof and Ratnayake, 2016; Dreher et al., 2007), only men were included in this study. Our behavioural findings seem to be in line with previous reports showing that acute stress disrupts reward learning in women (Berghorst et al., 2013; Bogdan et al., 2011; Bogdan and Pizzagalli, 2006; Morris and Rottenberg, 2015; Paret and Bublatzky, 2020), but further studies are needed to assess whether acute stress affects the same neurocomputational mechanisms of reinforcement learning in both men and women. Furthermore, given that our stress manipulation did not increase stress levels in all participants, future studies should explicitly account for individual differences and for the modulatory role of those individual differences on the neural mechanisms that underlie altered reinforcement learning under acute stress.

## 5 Conclusions

We present evidence that acute stress blunts prediction error signals in the dorsal striatum during reinforcement learning. This effect seems to be mostly driven by decreased positive prediction error signals, which might explain why individuals learn worse from the rewarding outcomes of their choices when under acute stress. Our findings are consistent with a neurobiological framework of stress-induced dopamine disruptions and can contribute to a better understanding of the neural mechanisms that underlie the deleterious impact of acute stress on reward learning. Ultimately, this study may offer important mechanistic insights into the impact of acute stress in everyday life as well as on designing appropriate interventions.

## CRediT authorship contribution statement

**Joana Carvalheiro:** Conceptualisation, Methodology, Software, Formal Analyses, Investigation, Writing – original draft, Writing - Review & Editing. **Vasco A. Conceição:** Conceptualisation, Writing - Review & Editing. **Ana Mesquita:** Conceptualisation, Writing - Review & Editing, Supervision. **Ana Seara-Cardoso:** Conceptualisation, Writing - Review & Editing, Supervision.

## Declaration of competing interest

The authors declare no competing interests.
